# Bioaccumulation of toxic and essential elements and enzymatic responses in native fish from the middle Tocantins River

**DOI:** 10.1038/s41598-026-39611-3

**Published:** 2026-03-08

**Authors:** Thiago Machado da Silva Acioly, José Iannacone, Karuane Saturnino da Silva Araújo, Jerusa Maria de Oliveira, Muhammad Ilyas, José Fábio França Orlanda, Diego Carvalho Viana

**Affiliations:** 1 Postgraduate in Animal Science (PPGCA/UEMA), Multi-User Laboratories in Postgraduate Research (LAMP), State University of Maranhão, São Luís, 65081-400 Brazil; 2 Federal Institute of Education, Science and Technology of the Sertão Pernambucano (IFSertãoPE), Campus Floresta, Floresta, Pernambuco 56400-000 Brazil; 3 Animal Ecology and Biodiversity Laboratory (LEBA), Universidad Nacional Federico Villarreal, 15007 Lima, Peru; 4 Carrera de Ingeniería Ambiental, Universidad Científica del Sur, Lima, Perú; 5 Animal Experimentation Laboratory, NexusBioTox, Campus of Engineering and Agricultural Sciences, Federal University of Alagoas, Maceió, Alagoas Brazil; 6 Department of Environmental Sciences, International Islamic University, Islamabad, 44000 Pakistan; 7 Center for Exact, Natural and Technological Sciences (CCENT), State University of the Tocantina Region of Maranhão (UEMASUL), Imperatriz, 65900-000 Brazil; 8 Center of Agrarian Sciences, Center for Advanced Morphophysiological Studies (NEMO), State University of the Tocantina Region of Maranhão (UEMASUL), Imperatriz, 65900-000 Brazil

**Keywords:** Food safety, Dietary intake, Heavy metals, Freshwater fish, Risk assessment, One health, Biochemistry, Ecology, Ecology, Environmental sciences, Zoology

## Abstract

This study provides the first ecotoxicological evaluation of potentially toxic elements (PTEs), essential elements (EEs), and enzymatic biomarkers in two native fish species, Branquinha (*Psectrogaster amazonica*) and Branquinha-cascuda (*Caenotropus labyrhinthicus*), from the middle Tocantins River, Brazil. Specimens were collected from the urban riverside zone of Beira Rio (P1) and a fluvial beach near the rural community of Embiral (P2). PTEs and EEs concentrations were measured in liver and muscle tissues, and biochemical biomarkers included acetylcholinesterase (AChE), aspartate aminotransferase (AST), alanine aminotransferase (ALT), and alkaline phosphatase (ALP). Human health risks associated with fish consumption were assessed using the bioconcentration factor (BCF), risk quotient (RQ), and estimated daily intake (EDI). In *P. amazonica* (urban zone), arsenic (As) exceeded limits by 83–266% in muscle and 60–220% in liver, lead (Pb) in liver by 95–1850%, and selenium (Se) by up to 4537%. In *C. labyrhinthicus* (rural zone), As and Se in muscle exceeded limits by 185–470% and 5–200%, respectively, while Pb and zinc (Zn) were not detected. BCF values indicated moderate bioaccumulation of sulfur (S) (2867.29) and iron (Fe) (2641.30). Risk assessment revealed a high level of concern, with an RQ for Se reaching 53.13 and As intake exceeding child safety thresholds sevenfold, highlighting significant food safety risks.

**Supplementary Information:**

The online version contains supplementary material available at 10.1038/s41598-026-39611-3.

## Introduction

Environmental contamination from domestic effluents, agricultural practices, industrial waste, and urban expansion has emerged as a critical global issue, compromising the integrity of aquatic ecosystems and posing risks to organisms and human populations that depend on these environments^1,2^. Among the most hazardous elements in freshwater environments are arsenic (As), chromium (Cr), lead (Pb), cadmium (Cd), mercury (Hg), nickel (Ni), and zinc (Zn), which may become highly toxic when exceeding biological thresholds^3,4^. These contaminants can impair food webs and fish safety, triggering cytotoxic, mutagenic, reproductive, developmental, and carcinogenic effects in exposed organisms^5–7^. Fish occupy key ecological and nutritional roles and are recognized as effective bioindicators of aquatic pollution due to their physiological sensitivity, trophic position, and ability to accumulate contaminants throughout their life cycle^8,9^. Exposure occurs through multiple routes, including ingestion of contaminated food sources, branchial respiration, and dermal absorption resulting from contact with suspended particles and sediments^10,11^). Many contaminants, particularly trace metals such as As, Cd, Hg, and Pb, tend to accumulate in metabolically active organs like the liver and in muscle tissues consumed by humans, posing health risks even at low environmental concentrations^7,12,13^. In recognition of their toxicity, the International Agency for Research on Cancer (IARC) classifies As, Cd, and hexavalent Cr as Group 1 carcinogens (carcinogenic to humans), while Pb is categorized as Group 2B (possibly carcinogenic)^14^.

The bioaccumulation of potentially toxic metals in fish has been increasingly documented worldwide, reflecting a widespread environmental and public health concern. Rapid agricultural expansion, industrial effluent discharge, and urban and peri-urban runoff have contributed to the transport of these elements (including As, Pb, Cd, Hg, and Se) into freshwater systems, promoting their entry into the food chain^15–17^. Once accumulated in fish tissues, these elements can biomagnify, ultimately reaching human consumers. Chronic consumption of contaminated fish can lead to neurological impairments, hematological disturbances, developmental delays, carcinogenesis, gastrointestinal damage, cardiovascular disease, and elevated cancer risk, even when ingestion occurs in low but continuous doses^13,18,19^. Studies in Bangladesh^20–22^, Serbia^23^, the Pearl River Basin in China^24^, and the Orashi River in Nigeria^25^ consistently demonstrate that rivers affected by agroindustrial, mining, and urban pressures exhibit elevated metal concentrations in fish tissues, underscoring the universality of this issue across distinct geographic and socioeconomic contexts.

Despite these risks, fish remain an essential source of macro- and micronutrients, including proteins, iron (Fe), zinc (Zn), copper (Cu), Selenium (Se), and particularly long-chain omega-3 polyunsaturated fatty acids such as docosahexaenoic acid (DHA) and eicosapentaenoic acid (EPA). These compounds play key roles in maintaining cardiovascular and neurological functions and have been associated with reduced risks of coronary disease and inflammation modulation^26,27^. To minimize exposure-related risks, organizations such as the European Commission (EC), the world health organization (WHO), the food and agriculture organization (FAO), and the United States environmental protection agency (USEPA) have established maximum permissible limits and intake guidelines to evaluate dietary exposure risks for both general and vulnerable populations, including children^25^. These regulatory frameworks are essential for guiding risk assessments that support public health protection.

The Tocantins River is an important watercourse that flows through the states of Maranhão and Tocantins, Brazil, emptying into Pará, and forms part of the Araguaia-Tocantins system^28^. Located in the transitional zone between the Cerrado and Amazon biomes, it plays a vital strategic role both regionally and nationally, serving as a major transportation corridor and gateway to the Amazon region. Its surrounding areas host dynamic agribusiness and industrial activities that significantly contribute to economic development and job creation. The river averages 30 m in depth, 500 m in width, and has a discharge of approximately 13.600 m^3^/s^29^. Communities along its banks, as well as nearby villages, depend heavily on the river for domestic water use, recreation (such as bathing), and artisanal fishing, which supports both subsistence and local commerce^28^. However, environmental pressures such as agricultural expansion, monoculture practices (e.g., eucalyptus plantations), aquaculture, and inadequate urban sanitation infrastructure have contributed to the degradation of water quality^29–31^. Moreover, urban runoff can transport potentially toxic metals, oils, excess nutrients, pesticides, polycyclic aromatic hydrocarbons (PAHs), and emerging contaminants such as pharmaceutical metabolites into the river^32–36^. This situation is particularly concerning for local communities that rely on fishing as a traditional source of food and income^28^. Riverside families depending on water and fish from the middle Tocantins River face risks from contaminants, highlighting the urgent need for effective monitoring and management. This study provides the first comprehensive assessment of potentially toxic elements (PTEs) and essential elements (EEs) in the muscle and liver tissues of two native fish species, *Psectrogaster amazonica* Eigenmann & Eigenmann, 1889 and *Caenotropus labyrhinthicus*^37^, from the middle Tocantins River, addressing a notable gap in regional knowledge. These species are ecologically important and widely consumed by riverside communities, making them suitable sentinel organisms for monitoring contaminant exposure, as documented in previous ecological and socio-environmental studies^28,38,39^. To evaluate potential human health risks, the bioconcentration factor (BCF), risk quotient (RQ), risk index (RI), and estimated daily intake (EDI) were calculated. This integrated approach directly links environmental contamination to real dietary exposure in traditional Brazilian Amazonian riverine communities.

## Materials and methods

### Sampling sites

**Fig. 1:**
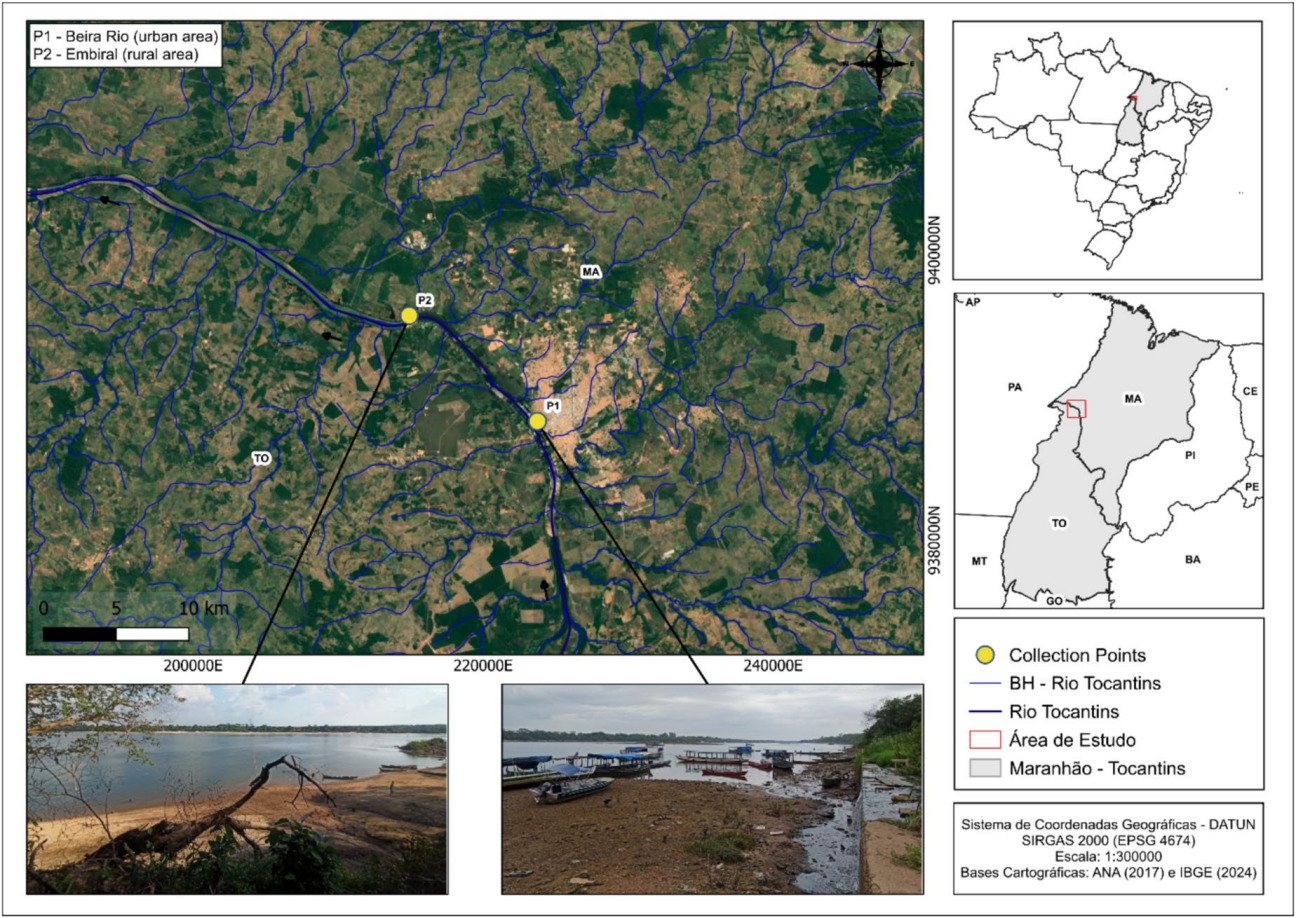
Location, main characteristics, and geographic coordinates of the sampling sites in the middle Tocantins River, Maranhão, Brazil.

The study was conducted in the middle stretch of the Tocantins River, within the area influenced by the municipality of Imperatriz (sub-basin 23), Maranhão, Brazil. Sampling campaigns took place during the hot and dry season of 2023 (July to December), according to the Köppen-Geiger climate classification (Köppen & Geiger, 1928). Located at the interface between the Brazilian savanna (Cerrado) and Amazon biomes, this river holds considerable regional and national relevance as a transportation corridor and gateway to the Amazon. Fish specimens were collected from two key sites: the urban riverside area known as “Beira Rio” (coordinates: 2°34′57.82"S; 44°21′34.92"W) (P1) and a fluvial beach near the rural community of Embiral (coordinates: 5°41′17.6658"S; 47°28′30.40752"W) (P2), approximately 40 km from the municipality of Cidelândia (Fig. 1). Sampling points were georeferenced in the field using a GARMIN 4215 GPS receiver. The map was created using QGIS software (version 3.40.9; https://qgis.org/) under the SIRGAS 2000 geographic coordinate system (EPSG:4674). Cartographic base layers were obtained from publicly available datasets provided by the Brazilian National Water Agency^40^ and the Brazilian Institute of Geography and Statistics^41^.

A recent study conducted in the middle Tocantins River, Maranhão, Brazil, offers a detailed environmental characterization of two distinct sampling sites. The first site (Beira Rio), located within an urban zone, is heavily influenced by anthropogenic activities, including boat traffic, recreational use, improper waste disposal, and discharge from iceboxes and untreated urban sewage. This area is notably impacted by high viral loads in sewage, reaching concentrations of 260 billion copies per liter^42^. The second site, located upstream from the urban area, lies near a pulp and paper industry and is subject to its effluent discharge, representing a potential point source of pollution. Despite this, the area is frequently used for recreational activities on river beaches and private estates and is also a site for small-scale artisanal fishing by residents. Previous studies conducted at four sites along the middle Tocantins River, Maranhão^29,43^, served as the basis for selecting the current sampling locations, representing an urban site under greater anthropogenic influence and a rural site under lower impact. This selection strategy aimed to minimize environmental disturbance and reduce the number of animals required for the study, in accordance with ethical research principles.

### Fish sampling

Fish specimens were captured using gill nets with mesh sizes ranging from 1.5 to 8.0 cm (measured between opposite knots), in addition to cast nets. A total of 30 individuals were collected, comprising 15 specimens of *P. amazonica* from the urban site (P1) and 15 specimens of *C. labyrhinthicus* from the rural site (P2). The use of different species at each site was based on local species availability, ecological representativeness, and relevance to human consumption^28,38,39^, as *P. amazonica* predominated at P1 and *C. labyrhinthicus* at P2. This approach is consistent with ecotoxicological and biomonitoring studies that employ locally abundant and site-specific fish species as sentinel organisms when paired sampling of the same species is not achievable^44^. Biometric data (total weight, g,standard length, mm were recorded using a portable scale and an ichthyometer, and sex was determined. Species identification was based on morphological characteristics, supported by taxonomic literature and the traditional ecological knowledge of local fishers.

Muscle and liver tissues were collected for the analysis of PTEs and EEs. Evisceration was performed in the field using sterile surgical scalpels, and samples were stored in polyethylene containers at − 4 °C for transport and at − 20 °C upon arrival at the laboratory at the State University of the Tocantina Region of Maranhão (UEMASUL) until analysis. Anesthesia and euthanasia followed internationally and nationally recognized ethical guidelines. Eugenol was used as the primary anesthetic, with auxiliary ice slurry to reduce metabolic activity, followed by anesthetic overdose and spinal cord sectioning, in accordance with the AVMA Guidelines for the Euthanasia of Animals^45^ and the Brazilian Guidelines for Best Practices in Animal Euthanasia^46^.

### Preparation and chemical processing of fish samples

To determine the total concentration of elements in fish tissues, approximately 0.5 g of dried muscle tissue was accurately weighed and digested using an acid mixture composed of 1 mL of hydrochloric acid (HCl) (50%), 1 mL of nitric acid (HNO_3_) (50%), and 8 mL of deionized water. The procedure followed the guidelines outlined in Method 3050B by the U.S. Environmental Protection Agency^47^. The digestion was conducted using a heating block (ECO 16, VELP Scientific) at 120 °C for 2 h. Subsequently, the quantification of PTEs, Al (aluminum), As (arsenic), Au (gold), Sb (antimony), Cd (cadmium), Cr (chromium), Pb (lead), Ni (nickel), Sr (strontium), Ti (titanium), and In (indium),and EEs, B (boron), Ba (barium), Be (beryllium), Ca (calcium), Co (cobalt), Cu (copper), Fe (iron), K (potassium), Mg (magnesium), Mn (manganese), Na (sodium), P (phosphorus), S (sulfur), Se (selenium), Si (silicon), Sn (tin), and Zn (zinc) was carried out using inductively coupled plasma optical emission spectrometry (ICP-OES) with a SHIMADZU ICPE-9000 (Kyoto, Japan). All measurements were performed in triplicate, and results were expressed in mg/kg of fish tissue, as calculated using Eq. (1)^48^. Elements that were below detection limits or occurred at very low concentrations were retained in the analytical protocol but are not discussed in detail in the Results section due to their limited ecological or toxicological relevance. The measured concentrations were then evaluated against established national and international regulatory standards to assess compliance and potential environmental risk (Table 1).

1 $${\text{Concentration of the metal }}({\mathrm{mg}} / {\mathrm{kg}}){ } = \frac{{{\text{(Instrument reading }} \times {\text{ volume of the extract) }}}}{{\text{mass of fish digested}}}$$

**Table 1:** Guidelines for element concentrations (mg/kg) in edible fish tissues.

Elements	Brazil	Mercosul (GMC/RES. Nº 12/11)	Chile	FAO (n◦ 66/2003)	WHO/FAO (CODEX)
As	1.0	1.0	1.0		0.5
Cd	1.0	0.05		0.05	0.5
Cu			10		
Se			0.3		
Pb	2.0	0.3	2	0.2	0.3
Zn			100		

Brazil^49^. MERCOSUL^50^. Chile^51^. FAO^52^. WHO/FAO Codex Alimentarius Commission^53^.

To ensure the reliability of elemental analyses in fish tissues, rigorous quality assurance (QA) and quality control (QC) procedures were implemented following the U.S. EPA quality management system^54^ and the internal standards of the analytical laboratory. These procedures included: (a) strict adherence to equipment manufacturers’ specifications, (b) regular instrument calibration, (c) proper sample handling with documented custody and storage, (d) standardized procedures before and after sample collection, and (e) comprehensive documentation of all analytical steps. Only analytical-grade reagents and chemicals (Merck, PA grade) were used throughout the study^55^^-56^. Multi-element ICP standards (Merck), calibrated according to DKD-accredited procedures (DKD-K-14302), were used for external calibration curves. Instrument calibration was performed prior to analysis and routinely verified throughout analytical runs, with recalibration conducted whenever necessary, in accordance with manufacturer specifications. All calibration curves exhibited high linearity, with correlation coefficients (r) exceeding 0.998. Method performance was evaluated through linearity, precision, and recovery. Precision was assessed by replicate analyses, while recovery was determined using matrix spike experiments, in which known concentrations of multi-element standards were added to fish tissue samples prior to analysis. Recovery rates ranged from 90 to 109%, confirming satisfactory analytical accuracy and reliability. No certified reference material (CRM) specific to fish tissues from the Tocantins River or to the studied species was available at the time of analysis, which represents a limitation of the study. However, this work constitutes the first ecotoxicological assessment of elemental contamination in these species and this river section, and data quality was supported by consistent calibration linearity, acceptable recovery rates, and reproducible analytical performance.

### Fulton condition factor (Q)

The Fulton condition factor (Q), also known as the condition index, was used to assess the length–weight relationship of the fish samples and their condition in the river according to Eq. (2)^57^. This index provides an estimate of the nutritional status, health, and well-being of the fish, and is useful for understanding how the physical condition of the fish can vary with environmental and nutritional factors.

2 $${\text{Q = }}\frac{{\mathrm{W}}}{{{\mathrm{L}}^{{3}} }} \times {100}$$

where W is the total body weight of the fish in grams (g), and L is the total length of the fish in centimeters (cm). According to Sekitar et al.^58^ and Datta et al.^59^, a Q ≤ 1 indicates poor condition (possibly due to factors such as lack of food, environmental stress, or disease), a Q = 1.2 indicates moderate condition (expected for the length and weight), and a Q ≥ 1.4 indicates proportionally good condition (good health status). This index is widely used in ecological and fisheries biology studies to monitor the conditions of different fish populations and to better understand the impacts of environmental factors on these populations^48,60^.

### Bioconcentration factor (BCF)

The BCF quantifies how readily a compound accumulates in aquatic organisms relative to its concentration in the surrounding water, as expressed in Eq. (3)^61^. This parameter is calculated by dividing the concentration of the compound in the organism by its concentration in the aquatic environment, with water concentration data in this case sourced from Acioly et al.^28^. The BCF is a critical metric for evaluating the ecological and health risks associated with contaminants of emerging concern^62,63^, and can be determined in various organs or tissues such as liver, muscle, kidney, or brain^64^.

3 $${\mathrm{B}}\text{CF } = \frac{\text{concentration of the substance in the body} }{\text{concentration of the substance in water}}$$

Fish are commonly used in BCF assessments due to their ecological and nutritional significance, as well as the availability of standardized test methods. According to the classification proposed by the US EPA^65^, compounds with a BCF below 1000 are considered not bioaccumulative, those between 1000 and 5000 are classified as bioaccumulative, and values exceeding 5000 are indicative of high bioaccumulation potential. Thereby, a BCF of 1000 means that the organism concentrates the product up to a value a thousand times higher than that of the environment^66^.

### Risk assessment for human health from fish consumption

Human health risk from fish consumption was evaluated using the risk quotient (RQ) and the cumulative risk index (RI). The RQ for each metal was calculated as the ratio between its concentration in fish muscle tissue and the corresponding maximum permissible limit (ML) established by Brazilian and international regulations^49,67^, as shown in Eq. (4). Values of RQ < 1 indicate no expected risk, whereas RQ ≥ 1 suggest potential adverse effects^65^.

4 $$\text{RQ } = \frac{\mathrm{C}}{{\mathrm{ML}}}$$

where C is the concentration of the metal in fish muscle (mg/kg, wet weight) and ML is the regulatory maximum limit (mg/kg). To assess the combined risk from multiple metals, the RI was calculated as the sum of individual RQ values (Eq. 5). Higher RI values indicate greater potential health concern from dietary exposure^68^.

5 $${\text{RI = }} \sum {\mathrm{RQi}}$$

where RQ_i_ represents the risk quotient of each metal i.

### Estimated daily intake (EDI)

To improve human health risk assessment, the EDI of each bioaccumulated metal in fish muscle tissue was determined by dividing its concentration by the average daily fish consumption rate, normalized by average adult body weight^69^. This evaluation followed the methodology proposed by the US EPA^7^, with adaptations described by Naji et al.^70^. Two fish consumption scenarios were considered: the national average for the Northern region of Brazil of 23 kg/person/year^71^ and the higher regional estimate of 151.98 kg/person/year for the Brazilian Amazon population^72,73^. Risk characterization and safe consumption thresholds were established according to the approach developed by Naji et al.^70^.

EDI was calculated using Eq. 6, where C is the concentration of metals in fish muscle (mg/kg), IR is the ingestion rate—63 g/day for the general Brazilian population^71^ and 416.39 g/day for individuals in the Amazon region^72,73^—and BW is body weight (70 kg for adults, 15 kg for children), based on standard reference values widely used in human health risk assessment^7,74–76^. The resulting EDI values were compared against reference doses (RfDs) defined by ANVISA^77^ for most elements, and by FAO/WHO^78^ for lead (Pb), which represent the maximum daily intake considered safe. Including both ingestion scenarios allows for a broader assessment of potential health risks and supports the development of consumption guidelines, especially for Amazonian populations with high fish consumption habits.

6 $$\text{EDI } = \frac{{\mathrm{C}} }{\raisebox{1ex}{${\mathrm{IR}}$}\!\left/ \!\raisebox{-1ex}{${\mathrm{BW}}$}\right.}$$

### Enzyme biomarkers assay

The liver and muscle samples were processed as follows: 100 mg of each tissue was weighed on an analytical balance and placed in a 1.5 ml glass test tube. Next, 0.4 ml of cold 1X phosphate-buffered saline (PBS) was added, and the tissue was homogenized using a micro-homogenizer. The tubes were centrifuged at 4.000 rpm for 15 min. The supernatant was transferred to microtubes and kept refrigerated (4 °C) until all analyses were completed. The activities of acetylcholinesterase (AChE), aspartate aminotransferase (AST), alanine aminotransferase (ALT), and alkaline phosphatase (ALP) were then measured using a spectrophotometer (Model 800 XI) with thermostatted cuvettes (37ºC).

#### Quantification of AChE in muscle and liver

AChE activity was determined using the Enzymatic Kinetic Test (Bioclin K094). The enzyme activity catalyzes the hydrolysis of butyrylthiocholine to thiocholine and butyrate; thiocholine reduces potassium hexacyanoferrate (III), a yellow compound, to colorless hexacyanoferrate (II). The decrease in absorbance is measured at 405 nm. The minimum detection limit is 50 U/L, and the test is linear up to 20.000 U/L.

For the assay, 10 µL of supernatant was added to a cuvette, followed by 400 µL of reaction buffer (pyrophosphate, potassium hexacyanoferrate (III), stabilizer, and preservative). The contents in the cuvette were mixed, homogenized, and incubated for approximately 3 min at 37 °C. Then, 100 µL of substrate (butyrylthiocholine, buffer, stabilizer, and preservative) was added. After homogenization, the mixture was incubated for exactly 2 min in a thermostated cuvette at 37 °C, and absorbance was measured at 405 nm. The readings were taken again after 1, 2, and 3 min. AChE activity was expressed as nmol/mg/min. The absorbance value was multiplied by 62.000 to obtain the value in U/I (international units). The linear equation obtained was Y = 1.0028X—170.85, with a correlation coefficient of 0.9964.

#### Quantification of AST in the liver

AST activity was determined using the Enzymatic Kinetic Test (Bioclin K048). AST catalyzes the transfer of amino groups from aspartate to α-ketoglutarate, forming glutamate and oxaloacetate. In the presence of malate dehydrogenase (MDH), oxaloacetate reacts with NADH, converting to malate and oxidizing NADH to NAD + . The rate of oxidation is proportional to the AST activity in the sample, in the absorbance readings taken at 340 nm. The minimum detection limit is 1.756 U/L, and the test is linear up to 400 U/L.

For the assay, 100 µL of supernatant was added to a cuvette, followed by 1 mL of working reagent (substrate + coenzyme). The contents in the cuvette were mixed, homogenized, and incubated for approximately 1 min at 37 °C. An initial absorbance reading was taken, with the timer started simultaneously. Subsequent readings were taken at 1, 2, and 3 min. The average differences in absorbance per minute (A/min) were calculated and used for the result calculation. AST activity was expressed as nmol/mg/min. The absorbance value was multiplied by 1.746 to obtain the value in U/I (international units). The linear equation obtained was Y = 0.9765X + 1.0283, with a linear correlation coefficient of 0.9999.

#### Quantification of ALT in the liver

ALT activity was determined using the Enzymatic Kinetic Test (Bioclin K049). In this assay, the kinetic determination of ALT follows this sequence: ALT catalyzes the transfer of the amino group from alanine to α-ketoglutarate, forming pyruvate and glutamate. Pyruvate, in the presence of lactate dehydrogenase (LDH), reacts with NADH, converting to lactate and oxidizing NADH to NAD + . The rate of oxidation is proportional to the ALT activity in the sample, in the absorbance readings taken at 340 nm. The minimum detection limit is 0.998 U/L, and the test is linear up to 400 U/L.

For the assay, 100 µL of supernatant was added to a cuvette, followed by 1 mL of working reagent (substrate + coenzyme). The contents in the cuvette were mixed, homogenized, and incubated for approximately 1 min at 37 °C. An initial absorbance reading was taken, with the timer started simultaneously. Subsequent readings were taken at 1, 2, and 3 min. The average differences in absorbance per minute (A/min) were calculated and used for the result calculation. ALT activity was expressed as nmol/mg/min. The absorbance value was multiplied by 1.746 to obtain the value in U/I (international units). The linear equation obtained was Y = 0.9693X + 1.33, with a correlation coefficient of 0.9995.

#### Quantification of ALP in liver and muscle

ALP activity was determined using the Enzymatic Kinetic Test (Bioclin K021). The methodology employed was the IFCC Kinetic method, where ALP catalyzes the transfer of the phosphate group from the substrate p-Nitrophenylphosphate (pNPP) to 2-Amino-2-Methyl-1-Propanol (AMP), forming p-Nitrophenol. The rate of p-Nitrophenol release, which has high absorbance at 405 nm, is proportional to the ALP activity in the sample. The minimum detection limit is 3.08 U/L, and the test is linear up to 1500 U/L.

For the assay, 20 µL of supernatant was added to a cuvette, followed by 1 mL of working reagent (buffer, p-Nitrophenylphosphate substrate, stabilizer, and preservative). The contents in the cuvette were mixed, homogenized, and incubated for approximately 1 min at 37 °C. An initial absorbance reading was taken, with the timer started simultaneously. Subsequent readings were taken at 1, 2, and 3 min. The average differences in absorbance per minute (A/min) were calculated and used for the result calculation. ALP activity was expressed as nmol/mg/min. The absorbance value was multiplied by 2,757 to obtain the value in U/L (international units). The linear equation obtained was Y = 0.9979X—0.486, with a correlation coefficient of 0.9996.

### Statistical analysis

Statistical analyses were conducted using PAST software, version 4.03 (2020), with the significance level set at p < 0.05. Data normality was assessed using the Kolmogorov–Smirnov and Shapiro–Wilk tests, and the homogeneity of variances was verified with Levene’s test. Since the data did not meet the assumptions of normality (Shapiro–Wilk, p < 0.05), differences in metal concentrations and enzymatic activities between sites (P1 and P2) were evaluated using the non-parametric Mann–Whitney U test. Correlation analyses (e.g., Pearson or Spearman) were not performed because only two sampling sites were available, which precludes meaningful estimation of inter-variable relationships. The association between two variables is expressed by the correlation coefficient r, ranging from − 1 to + 1, where values close to zero indicate no linear relationship and values approaching ± 1 indicate a perfect correlation^79^. Positive values of r (r > 0) indicate direct relationships between variables, whereas negative values (r < 0) indicate inverse relationships. The strength of the associations was interpreted following Akoglu^79^, considering |r|≤ 0.30 as weak, 0.40–0.60 as moderate, and |r|≥ 0.70 as strong.

### Ethical statement

This study was approved by the Ethics Committees on Animal Use of the State University of the Tocantins Region (CEUA No: 1025220722) and the State University of Maranhão (CEUA Nº: 040/2023), with collection authorized by SISBIO (Authorization N°: 87310–1). All experimental procedures involving animals were performed in accordance with relevant institutional, national, and international guidelines and regulations for the care and use of animals in research. This study is reported in accordance with the ARRIVE guidelines 2.0 for the reporting of animal research^80^.

## Results and discussion

Previous environmental assessments conducted in the middle Tocantins River basin provide essential context for the interpretation of the present findings. Acioly et al.^29^ performed an integrated evaluation of physicochemical and biological parameters in surface waters, including temperature, pH, turbidity, electrical conductivity, redox potential, dissolved oxygen, salinity, chlorophyll, total dissolved solids, nitrogenous compounds, and trace elements, thereby characterizing baseline ecosystem conditions. In a complementary study, Machado da Silva Acioly et al.^43^ investigated the same elements in bottom sediments using contamination and ecological risk indices, revealing marked spatial variability and clear signals of anthropogenic influence along different sectors of the basin. Together, these studies establish a robust environmental quality baseline for the middle Tocantins River. Building upon this environmental framework, the results of the present study provide biological evidence that such contamination is not restricted to abiotic compartments but is effectively transferred to aquatic biota. The detection of toxic elements such as As, Pb, and Se in fish tissues indicates that metals present in the environment are biologically available and capable of entering higher trophic levels. Moreover, differences in metal accumulation patterns between fish collected from urbanized and rural areas highlight the influence of localized anthropogenic pressures on contaminant exposure. Collectively, these findings establish a direct link between environmental contamination and biological uptake, providing the foundation for the subsequent discussion of bioaccumulation dynamics, physiological responses, and potential ecological and human health implications.

### Biometric data and the fulton condition (Q)

For *P. amazonica*, individual weights ranged from 83 to 110 g and standard lengths from 17 to 21 cm. *C. labyrhinthicus* individuals weighed 28–57 g with lengths of 10.8–13 cm (Table 2). The Q factor, a widely used indicator of fish health, indicated good body condition for both species (*P. amazonica*: Q = 1.40; *C. labyrhinthicus*: Q = 2.20). According to Datta et al.^59^, Q values above 1.4 typically reflect good physiological status, suggesting that the captured individuals were not under significant stress at the time of sampling. This apparent maintenance of good body condition, despite the presence of elevated metal concentrations, suggests sublethal exposure scenarios in which fish are able to sustain somatic growth while undergoing biochemical and physiological adjustments. Such a pattern indicates that condition indices alone may not fully capture underlying toxic stress.

**Table 2:** Taxonomy of biological material and biometric measurements.

Common name (scientific name)	Environment/ trophic level	Sampling point	Number of fish collected	Standard length (CP)		Weight (W)		Fulton condition factors (Q)
				Mean ± SD	Min–Max	Mean ± SD	Min–Max	
*P. amazonica*	d/ 2.2 and 2.79	P1	15	19 ± 1.72	17–21	96 ± 10.15	83–110	1.40
*C. labyrhinthicus*	d/ 2.2 and 2.79	P2	15	12 ± 0.68	10.8–13	38 ± 6.88	28–57	2.20

P1: Beira Rio (urban area). P2: Embiral (rural area). bp: benthopelagic, d: demersal, pn: pelagic-neritic, Trophic level: herbivores may have values of trophic level between 2.0 and 2.19; tc: consumers who consume ‘mainly animals’ (carnivores) may have trophic levels equal to or greater than 2.8; and fish which are partly herbivore and partly carnivore, i.e. omnivores which consume 'plants/detritus + animals’ may have trophic levels between 2.2 and 2.79.

### PTEs and EEs in branquinha (*P. amazonica*) (urban zone)

The highest concentrations of elements in muscle tissue of *P. amazonica* were Ca > K > Mg > Na > Fe > Zn > P, whereas in liver the order was Fe > Ca > K > Na > Mg > Zn > P (Table 3). Al, although not among the most abundant elements, is noteworthy due to its toxicological relevance, with concentrations of 7.19 mg/kg in muscle and 3.34 mg/kg in liver. These values are below the No Observed adverse effect level (NOAEL) of 30 mg/kg body weight/day established by FAO/WHO^77^, suggesting no immediate health risk. Nevertheless, the detection of aluminum in both muscle and liver tissues indicates continuous environmental exposure and bioavailability, which may contribute to cumulative effects when combined with other elements present in the system. Therefore, the absence of acute risk does not exclude potential long-term ecological or health implications. In contrast, Se was markedly elevated, reaching 9.30 mg/kg in muscle and 13.91 mg/kg in liver, exceeding the international safety limit of 0.3 mg/kg by approximately 3100% in muscle and 4537% in liver. Se intake in humans primarily occurs through dietary sources such as meat, cereals, and seafood. Excessive dietary intake can lead to selenosis, with symptoms including dermatologic and neurologic disorders, gastrointestinal disturbances, hepatotoxicity, fatigue, irritability, and mild neurological effects^81,82^. These findings highlight a significant health concern for both fish and consumers.

**Table 3:** Average, maximum, and minimum values of PTEs and EEs concentrations (mg/kg) in tissues of *P. amazonica* from the middle Tocantins River (P1: urban zone).

Elements (detection limit)	Minimum (mg/kg)		Maximum (mg/kg)		Average (mg/kg) ± standard deviation	
	Muscle	Liver	Muscle	Liver	Muscle	Liver
PTEs						
Al (0.014)	2.27	1.2	13.27	6.93	7.19** ± **4.49	3.34** ± **2.36
As (0.002)	1.06	0.96	2.75	1.99	1.83 ± 0.65*	1.6 ± 0.41*
Au (0.001)	0.35	0.62	1.31	7.44	0.71** ± **0.37	3.95** ± **2.71
Cr (0.003)	0.78	–	3.70	–	1.83** ± **1.15	–
In (0.033)	0.19	–	2.31	–	0.31** ± **0.17	–
Pb (0.012)	–	1.44	–	6.74	–	3.90 ± 2.53*
Ni (0.002)	1.85	1.76	3.18	3.20	2.53** ± **1.04	2.66** ± **0.59
EEs						
Ba (0.002)	0.13	0.13	0.48	0.36	0.31** ± **0.25	0.14** ± **0.18
Ca (0.007)	77.38	139.04	759.31	489.09	590.14** ± **132.36	394.70** ± **128.16
Co (0.003)	–	3.60	–	7.80	–	5.54** ± **2.01
Cu (0.003)	0.14	6.52	0.66	13.71	0.31** ± **0.2	9.56** ± **3.10
S (0.001)	274.54	168.36	368.13	274.54	298.79** ± **34.68	210.57** ± **46.19
Se (0.024)	5.24	8.51	15.62	17.93	9.30 ± 4.13*	13.91 ± 3.92*
Sb (0.003)	1.23	1.64	2.78	4.09	1.94** ± **0.59	3.12** ± **1
Sn (0.021)	5.16	4.31	10.60	7.40	6.97** ± **2.2	6.15** ± **1.29
Si (0.015)	6.34	11.40	14.98	19.64	9.44** ± **3	15.04** ± **3.39
Fe (0.002)	7.64	432.88	20.92	2189.8	11.07** ± **5.1	1215.93** ± **703.1
Mg (0.001)	169.09	142.02	249.19	163	212.47** ± **32.12	150.45** ± **9.22
Mn (0.001)	0.18	1.11	3.55	1.62	1.04** ± **1.28	1.47** ± **0.21
Na (0.020)	163.76	149.90	229.39	305.07	186.90** ± **24.31	202.42** ± **73.23
P (0.004))	0.89	12.95	6.40	22.54	2.67** ± **2.01	17.33** ± **3.70
K (0.018)	207.45	280.22	284.86	572.73	257.33** ± **26.53	384.88** ± **110.41
Zn (0.002)	0.085	17.04	9.30	26.73	3.99** ± **4.25	21.72** ± **3.52

*Elements that are above national or/and international standards.

As concentrations in *P. amazonica* exceeded recommended limits, with 1.83 mg/kg in muscle (83–266% above Brazil/MERCOSUL and WHO/FAO limits, respectively) and 1.60 mg/kg in liver (60–220% above limits). Pb in liver (3.90 mg/kg) surpassed thresholds by 95% (Brazil/Chile), 1200% (MERCOSUL/WHO), and 1850% (FAO). These results indicate site-specific contamination and underscore the liver’s key role in bioaccumulation and detoxification of toxic elements. Zn was detected only in specimens from this area, with elevated hepatic levels, suggesting significant environmental exposure. The presence of As in muscle tissue raises concerns for food safety and human health. As, a well-known toxicant, is associated with gastrointestinal, neurological, cardiovascular, and hematological disorders^19,83,84^, reinforcing the need for ongoing environmental monitoring in urbanized and ecologically sensitive regions^50^.

Exposure to Pb represents a significant public health concern, particularly for populations consuming contaminated fish^27,85^. Pb is persistent, non-biodegradable, and one of the most prevalent aquatic contaminants. It acts as an immunotoxicant and is associated with various physiological, biochemical, and neurological disorders in humans^86,87^. In fish, Pb exposure affects reproduction, growth, and behavior^88–90^. Its hepatotoxic effects are primarily linked to oxidative stress, including excessive reactive oxygen species (ROS) generation, which can disrupt antioxidant enzyme activity and lead to cell death.

### PTEs and EEs in tissues of branquinha-cascuda (*C. labyrhinthicus*) (rural zone)

In muscle tissue, the most abundant elements were Ca > K > Mg > Na > Fe > P, whereas in liver the order was Fe > Ca > K > Na > Mg > P (Table 4). As and Se were detected in muscle tissue, with As at 2.85 mg/kg (185–470% above Brazil/MERCOSUL and WHO/FAO limits, respectively) and Se at 15.94 mg/kg (5180% above the international safety limit). Pb and Zn were not detected in rural specimens, suggesting lower local contamination. Nevertheless, the elevated As and Se concentrations indicate the need for ongoing environmental monitoring to safeguard both aquatic ecosystems and human health. The occurrence of elevated As and Se in fish from a rural setting indicates that contamination is not restricted to urban effluents. However, may also reflect upstream transport, natural geochemical background, or diffuse anthropogenic inputs. This reinforces that rural areas are not necessarily free from contamination risks.

**Table 4:** Average, maximum, and minimum values of PTEs and EEs concentration (mg/kg) in tissues of *C. labyrhinthicus* from the middle Tocantins River (P2: rural zone).

Elements (detection limit)	Minimum (mg/kg)		Maximum (mg/kg)		Average (mg/kg) ± standard deviation	
	Muscle	Liver	Muscle	Liver	Muscle	Liver
PTEs						
Al (0.014)	5.27	0.58	19.60	3.52	13.06** ± **3.99	1.82** ± **1.24
As (0.002)	0.30	–	6.70	–	2.85 ± 0.70*	–
Au (0.001)	0.66	0.004	4.25	1.23	1.26** ± **0.27	0.39** ± **0.48
Cr (0.003)	0.31	–	12.85	–	3.61** ± **3.22	–
In (0.033)	0.10	–	4.30	–	2.33** ± **1.19	–
Pb (0.012)	–	–	–	–	–	–
Ni (0.002)	0.37	–	6.56	–	3.32** ± **1.04	–
EEs						
Ba (0.002)	0.22	–	11.82	–	1.92** ± **0.61	–
Ca (0.007)	951.68	113.92	87,608.17	265.08	20,757.3** ± **11,173.31	183.52** ± **55.35
Co (0.003)	–	–	–	–	–	–
Cu (0.003)	0.16	0.37	1.9	2.60	0.79** ± **0.5	0.83** ± **0.81
S (0.001)	281.74	23.94	536.63	95.24	401.42** ± **73.46	44.15** ± **24.72
Se (0.024)	9.98	–	46.59	–	15.94 ± 10.55*	–
Sb (0.003)	1.62	–	6.94	–	3.12** ± **1.39	–
Sn (0.021)	8.24	–	37.93	–	12.19** ± **8.74	–
Si (0.015)	6.98	8.71	14.40	13.72	9.47** ± **2.74	10.65** ± **1.99
Fe (0.002)	1.65	30.22	18.5	683.70	12.11** ± **4.06	229.66** ± **230.99
Mg (0.001)	32.78	11.01	496.50	36.26	258.76** ± **104.69	18.54** ± **9.15
Mn (0.001)	0.04	0.14	4.22	0.58	1.29** ± **1.12	0.35** ± **0.16
Na (0.020)	25.99	42.82	631.91	91.74	173.21** ± **58.57	67.90** ± **18.14
P (0.004))	3.66	–	9.21	–	4.85** ± **1.89	–
K (0.018)	138.49	30.22	694.83	685.56	470.27** ± **176.74	230.35** ± **231.09
Zn (0.002)	1.65	0.16	3.94	28.20	11.86** ± **8.55	1.37** ± **1.22

*Elements that are above national or/and international standards.

In fish from water bodies in Macapá, concentrations of Pb, Cr, Ni, Hg, Cu, and Zn remained below Brazilian maximum limits (ML), indicating low risk to consumers^69^. However, Cd levels exceeded the legal threshold of 0.05 mg/kg, with average concentrations of ~ 0.06 (20% above the limit) and peak values reaching 0.09 in *Acaronia nassa* (Heckel, 1840) (80% above the limit). Other species, including *Acestrorhynchus altus*^91^, *Leporinus friderici*^92^, *Serrasalmus spilopleura*^14^ and *Pygocentrus nattereri*^14^, showed Cd concentrations of 0.07 (40% above the limit). These findings indicate that all sampled species are potentially unsafe for human consumption due to Cd contamination, regardless of size, weight, foraging behavior, or urban aquatic environment. Similarly, in fish from the lower Araguari River (Amapá), both Cd and Pb in muscle tissues exceeded national MLs, posing cumulative health risks from exposure to metal mixtures^68^. These findings are consistent with the pattern observed in the middle Tocantins River and indicate that metal contamination in Amazonian River systems arises from spatially distributed anthropogenic pressures rather than from isolated point sources.

The contamination observed in these urban areas reflects a broader environmental concern across the Amazon. It is estimated that nearly 90% of wastewater from urban zones in this region is discharged without treatment, introducing various pollutants, including potentially toxic metals, into rivers and tributaries^93^. A similar context is observed in the middle Tocantins River region, where this study was conducted. The local riverside population (96%) relies on native fish for food, 20% consume untreated river water, and 84% lack access to basic sanitation infrastructure, highlighting significant public health vulnerabilities^28^. Under such socio-environmental conditions, dietary exposure through fish consumption becomes a primary pathway for chronic metal intake, amplifying health risks in communities with limited access to sanitation and alternative protein sources. The spatial differences observed between sampling sites should not be interpreted as a strict control–impact contrast, but rather as a gradient of anthropogenic pressure along the middle Tocantins River. Both sampling areas are influenced, to varying degrees, by human activities, and the differences in metal accumulation in fish patterns reflect variations in land use, urbanization intensity, and proximity to potential contamination sources. The urban area is characterized by higher population density and greater exposure to industrial and domestic effluents, which may contribute to increased metal inputs into the aquatic system. In contrast, although the rural area is less urbanized, it is not free from anthropogenic influence, as agricultural activities, diffuse runoff, and changes in land use can also act as sources of metal contamination. This gradient-based interpretation provides a more ecologically realistic framework for understanding spatial variability in metal bioavailability and supports the subsequent analysis of bioaccumulation dynamics and physiological responses in fish.

### Bioconcentration factor (BCF)

In the muscle of *P. amazonica*, S showed the highest BCF (1757.59), followed by K (536.10) and Na (203.15), indicating significant accumulation (Table 5). In the liver, the element with the highest BCF was Fe (2641.30), suggesting its higher concentration in this organ. No element exceeded the 5000 thresholds, but some, such as sulfur, fall within the moderate bioaccumulation range (BCF between 1000 and 5000). In and Si had low BCFs (< 10), suggesting limited accumulation. For *C. labyrhinthicus*, in the muscle, S also had the highest BCF (2867.29), followed by Ca (1648.62) and K (723.49). In the liver, K (354.38) dominated, with a significant BCF, reflecting its higher concentration in this organ. The bioaccumulation of macronutrients remained predominant, but In and Cu showed low values, indicating limited accumulation (Table 5). The predominance of higher BCF values for EEs reflects their metabolic demand and physiological regulation, whereas the accumulation of certain PTEs in specific tissues highlights the role of organ function in modulating exposure and detoxification processes.

**Table 5:** Bioconcentration factor (BCF) of PTEs and EEs in the middle Tocantins fish.

Elements	Branquinha* (P. amazonica)* (urban zone)		Branquinha–cascuda *(C. labyrhinthicus)* (rural zone)	
	*BCF muscle*	BCF Liver	*BCF muscle*	BCF liver
PTEs				
Al	10.42	*4.84*	20.71	2.89
Au	8.88	49.38	12.60	3.90
In	0.53	–	4.57	–
EEs				
Ca	71.63	47.91	**1648.62**	14.58
Cu	6.20	191.20	15.80	16.60
Fe	24.07	**2641.30**	11.87	225.16
Na	203.15	219.15	151.06	59.56
K	536.10	801.83	723.49	354.38
S	**1757.59**	**1239.82**	**2867.29**	315.36
Se	46.50	69.55	93.76	–
Sn	*25.81*	22.78	35.85	–
Si	*2.12*	3.37	1.36	1.53

BCF < 1000 is non-bioaccumulative, 1000–5000 is bioaccumulative, and > 5000 indicates high bioaccumulation potential^65^.

### Human health risk assessment from fish consumption

There is a potential risk to human health associated with the consumption of the analyzed fish species, particularly due to elevated levels of Se and As. Se exhibited the highest RQ values across all tissues, with notable levels in the liver of *P. amazonica* (RQ = 49.16) and the muscle of *C. labyrhinthicus* (RQ = 53.13), exceeding the Brazilian regulatory limit of 0.3 mg/kg by more than 150 times (Table 6). It is important to note that selenium is an essential micronutrient and may reflect both environmental availability and physiological regulation related to homeostasis and detoxification, including protective interactions with other metals^94,95^. Consistent with this interpretation, the normal Q factor suggests that elevated Se levels may partly represent an adaptive response in fish,however, concentrations exceeding food safety limits remain a concern for human consumption. These results indicate a potential for adverse effects^65^ in frequent consumers. As also exceeded the legal threshold of 1.0 mg/kg in the muscle tissue of *P. amazonica* (1.83), yielding an RQ of 1.83 and representing an additional health concern.

**Table 6:** Risk quotients (RQ) and cumulative risk index (RI) of studied elements in muscle and liver tissues of fish species from the middle Tocantins River.

Elements	Branquinha *(P. amazonica)* (urban zone)		Branquinha–cascuda *(C. labyrhinthicus)* (rural zone)	
	*RQ muscle*	RQ liver	*RQ muscle*	RQ liver
Potentially toxic elements				
*As*	1.83*	*–*	–	–
*Pb*	–	1.95*	–	–
Essential elements				
*Cu*	0.03	0.96	0.08	0.08
*Se*	31.00*	46.37*	53.13*	–
RI	32.86	49.16	53.21	47.20

RQ < 1 indicates no risk to human health; *RQ > 1 suggests potential adverse effects^65^. RI values below 150 indicate low risk, while values between 150 and 300 suggest moderate risk^96^.

Although it was only detected in the urban zone with *P. amazonica*, its RI was low, suggesting minimal immediate risk. Selenium, however, was the primary contributor to the elevated RQ values in both muscle and liver tissues, indicating that its concentration, despite being an essential element, can reach concerning levels. Pb, found in the liver of *P. amazonica*, may not pose a direct risk to human health, as liver is not typically consumed. However, even at low concentrations in aquatic environments, Pb poses a significant threat to aquatic organisms due to its toxicity and bioaccumulation potential, which can harm biodiversity and affect local fisheries^69,97^. Cu showed RQs well below the threshold, indicating no significant risk. The results highlight the importance of monitoring Se and Pb concentrations and implementing fish consumption guidelines to protect public health. Taken together, these results indicate that health risks are driven not by a single contaminant, but by the combined presence of multiple elements^98,99^, particularly those exceeding regulatory limits in edible tissues.

The EDI assessment demonstrated that while Al, Cr, Ni, Fe, Mn, Cu, and Zn concentrations remained below reference doses (EDI < RfD) for both adults (Table 7) and children (Table 8)^77,78^, arsenic presented concerning exposure levels. In Scenario 2 (Amazonian consumption: 416.39 g/day), arsenic EDI values exceeded the FAO/WHO RfD by sevenfold for children (15 kg BW) versus 1.6-fold for adults (70 kg BW), revealing children experience 4.6 × greater exposure per kg body weight. This disproportionate risk for pediatric populations stems from their lower body mass and higher metabolic susceptibility to toxic elements. These health effects are dose-dependent and cumulative, meaning that even moderate daily intake can become hazardous over time^100–102^. The findings underscore an urgent need for: (1) species-specific consumption guidelines prioritizing low-As fish, particularly for vulnerable groups, (2) targeted monitoring programs in high-intake regions; and (3) community education about differential risks between adult and child consumption patterns.

**Table 7:** Estimated daily intake (EDI, mg/kg/bw day) of metals for adults (70 kg body weight) through consumption of fish muscle tissue from species collected in the middle Tocantins River, Maranhão, Brazil.

Fish species	EDI (adult)								
	Scenario	Al	As	Cr	Ni	Fe	Mn	Cu	Zn
Branquinha *(P. amazonica)* (urban zone)	1	0.0065	0.0016	0.0016	*0.0023*	0.0100	0.0009	0.0003	0.0036
	2	0.0428	0.0109	0.0109	*0.0150*	0.0658	0.0062	0.0018	0.0237
Branquinha–cascuda *(C. labyrhinthicus)* (rural zone)	1	0.0118	0.0026	0.0033	0.0030	0.0109	0.0012	0.0007	–
	2	0.0777	0.0170	0.0215	0.0197	0.0720	0.0077	0.0047	–
RfD (mg/kg/day)		0.286	0.003 (revoked)	0.045	1	3.47	2.3	6.935	23.5

Scenario 1: 63 g/day for the general Brazilian population^71^. Scenario 2: 416.39 g/day for individuals in the Amazon region^72,73^. RfD (Reference dose): ANVISA^77^, FAO/WHO^78^. Body weight (70 kg for adults, 15 kg for children) is based on standard reference values widely used in human health risk assessment^7,74–76^.

**Table 8:** Estimated daily intake (EDI, mg/kg/bw day) of metals for children (15 kg body weight) through consumption of fish muscle tissue from species collected in the middle Tocantins River, Maranhão, Brazil.

Fish species	EDI (children)								
	Scenario	Al	As	Cr	Ni	Fe	Mn	Cu	Zn
Branquinha* (P. amazonica)* (urban zone)	1	0.008	0.001	0.0005	*0.002*	0.015	0.0007	0.0003	0.004
	2	0.053	0.007	0.003	*0.013*	0.099	0.005	0.002	0.026
Branquinha–cascuda *(C. labyrhinthicus)* (rural zone)	1	0.055	0.012	0.015	0.014	0.051	0.005	0.003	–
	2	0.363	0.079	0.100	0.092	0.336	0.036	0.021	–
RfD (mg/kg/day)		0.286	0.003 (revoked)	0.045	1	3.47	2.3	6.935	23.5

Scenario 1: 63 g/day for the general Brazilian population^71^. Scenario 2: 416.39 g/day for individuals in the Amazon region^72,73^. RfD (Reference dose): ANVISA^77^, FAO/WHO^78^. Body weight (70 kg for adults, 15 kg for children) is based on standard reference values widely used in human health risk assessment^7,74–76^.

Evidence from human health risk assessments based on the consumption of contaminated fish strongly supports these concerns. Numerous studies worldwide have shown that arsenic concentrations in edible fish tissues frequently exceed regulatory limits and pose both non-carcinogenic and carcinogenic risks, particularly for populations with high fish intake^103,104^. For example, investigations in riverine and marine systems across South America have reported HQ and EDI values for arsenic above national and international safety thresholds, indicating increased lifetime cancer risk as well as potential hepatic, neurological, and cardiovascular effects in frequent consumers^105–108^. Consistent with these reports, our results show that arsenic intake from fish consumption in the middle Tocantins River exceeds safe limits, particularly under high-consumption scenarios, with children exhibiting exposure levels several-fold higher than adults. Collectively, these findings demonstrate that chronic dietary exposure to arsenic via contaminated fish represents a tangible public health risk and underscore the need for site-specific consumption advisories, long-term biomonitoring, and mitigation strategies for vulnerable riverine populations.

### Enzymatic biomarkers

The enzymatic responses evaluated in this study, AChE, CAT, GST, and related biomarkers, represent sublethal physiological responses to contaminant exposure. Alterations in the activity of these enzymes reflect adaptive adjustments in detoxification, antioxidant defense, and neurophysiological regulation pathways, allowing the early detection of environmental stress before the onset of clinical or population-level effects. Similar biomarker-based responses have been widely reported in aquatic and semi-aquatic organisms exposed to environmental contaminants, including neotropical fish species subjected to pesticides and metal mixtures^1,109,110^. In the present study, the modulation of enzymatic biomarkers is consistent with the bioaccumulation of toxic elements observed in fish tissues and with the simultaneous exposure to multiple metals, which may act additively or synergistically on redox balance and metabolic homeostasis^111–113^. Thus, the observed biomarker patterns provide functional evidence of physiological stress linked to metal exposure, and complement the chemical data by demonstrating biologically relevant effects in fish from the middle Tocantins River.

The biochemical responses presented in Table 9 reveal significant site- and tissue-specific variations in enzymatic activities and should not be interpreted as direct quantitative comparisons among distinct species. In *P. amazonica* from the urban site (P1), AChE activity was elevated in both muscle and liver when compared with basal values reported for Characiformes under non-impacted conditions. In contrast, *C. labyrhinthicus* from the rural site (P2) exhibited a marked reduction in muscle AChE activity. It is important to emphasize that phylogenetic, physiological, and ontogenetic differences among species, including variation in body size, metabolic rate, and the relative expression of cholinesterase isoforms, preclude direct interspecific comparisons of absolute AChE activity. Accordingly, interpretations were based on qualitative patterns of enzymatic responses within each site, rather than on cross-species numerical differences. The observed enzymatic alterations, even in individuals with normal condition factors, demonstrate that metal exposure is biologically active and capable of inducing functional responses before the onset of overt physiological impairment.

**Table 9:** Average, maximum, and minimum values of some enzymes in the muscle and liver tissue (U/I) of *P. amazonica* (urban zone) and *C. labyrhinthicus* (rural zone) from the middle Tocantins River.

Species	Enzyme	Minimum (U/I)		Maximum (U/I)		Average (U/I) ± standard deviation	
		Muscle	Liver	Muscle	Liver	Muscle	Liver
*P. amazonica* (urban zone)	AChE	12263.87	12263.87	38376.78	18481.23	19207 ± 9794	15372.55 ± 3405
	AST	–	191.29	–	410.22	–	294.39 ± 88
	ALT	–	94.41	–	822.14	–	528.89 ± 260
	ALP	18.77	384.68	274.64	604.78	121.72 ± 96	471.13 ± 74
*C. labyrhinthicus* (rural zone)	AChE	1818.71	1818.71	3994.78	3994.78	2544 ± 1071	2352.36 ± 991
	AST	–	115.26	–	430.38	–	290.02 ± 152
	ALT	–	35.18	–	41.95	–	38.28 ± 3
	ALP	82.05	82.05	170.09	170.09	89.39 ± 25	104.06 ± 40

*AchE* acetylcholinesterase, *AST* aspartate aminotransferase, *ALT* alanine aminotransferase, *ALP* alkaline phosphatase.

The increased AChE activity observed in *P. amazonica* at the urban site (P1) is consistent with a compensatory or adaptive biochemical response to chronic exposure to metals such as As and Pb, which are characteristic of urban effluents and surface runoff. Experimental and field studies indicate that prolonged sublethal exposure to neurotoxicants may elicit an initial upregulation of AChE activity as a mechanism to sustain cholinergic neurotransmission, frequently preceding enzymatic inhibition under more advanced or prolonged toxic stress^114–116^. Conversely, the reduction of AChE activity in *C. labyrhinthicus* from the rural site (P2) suggests subclinical neurotoxic inhibition, compatible with arsenic exposure even at comparatively lower environmental concentrations. A reduction in AChE activity are widely recognized as early biomarkers of neurotoxicity in Neotropical fish species, reflecting functional impairment of cholinergic signaling prior to overt behavioral or histopathological alterations^91,92,117^. Additionally, the analytical method employed (Bioclin K094) may present partial cross-reactivity with butyrylcholinesterase, potentially amplifying interspecific variability. This methodological constraint further supports the avoidance of absolute interspecies comparisons and reinforces the focus on site-specific enzymatic trends.

Elevated hepatic ALT and AST activities in *P. amazonica* indicate hepatocellular stress likely associated with higher bioaccumulation of Pb and As at the urban site, consistent with the liver’s central role in metal detoxification and biotransformation^118–120^. Moreover, experimental studies demonstrate that combined exposure to metals, particularly lead and arsenic, markedly enhances oxidative stress and hepatotoxicity compared to single-metal exposure^121^. The concomitant increase in ALP activity reflects non-specific physiological stress associated with chronic sublethal contamination. In *C. labyrhinthicus* from the rural site, lower and more stable enzymatic activities suggest reduced systemic biochemical stress, although the inhibition of AChE indicates that neurophysiological effects may occur even under diffuse or low-intensity contamination scenarios. When integrated with concentrations of PTEs and human health risk metrics (EDI and RQ), these enzymatic biomarkers provide early, sensitive, and biologically meaningful indicators of environmental degradation in the middle Tocantins River.

In addition to chemical contamination, the observed alterations in biochemical biomarkers indicate that metal exposure is biologically active, inducing increases in AST, ALT, and ALP and reductions in AChE activity. The integration of chemical and biochemical biomarkers with human health risk assessment strengthens the ecological relevance of the findings and enhances their translational value for public health. These results are directly relevant to environmental management and food safety policies and align with the United Nations sustainable development goals (SDGs), particularly SDG 3 (Good Health and Well-being), SDG 6 (Clean Water and Sanitation), SDG 14 (Life Below Water), and SDG 15 (Life on Land)^122^. Framed within a One Health perspective, this study highlights the interconnectedness of environmental quality, fish health, and human well-being.

### Correlation coefficients

Correlation analysis revealed several relevant associations among metal concentrations and biomarker responses (Figure S1). Strong positive correlations were observed between Fe and Al (r = 0.6985, p < 0.05; the same significance level applies to all correlations reported below) and between Mn and Zn (r = 0.7112), suggesting common geochemical or environmental sources, possibly linked to lithogenic inputs or sediment resuspension. A moderate positive correlation between Cu and Ni (r = 0.5749) indicates potential overlap in contamination sources, such as industrial effluents or agricultural runoff. Regarding enzymatic biomarkers, AChE activity showed a moderate negative correlation with hepatic Fe concentrations (r = − 0.6234), suggesting possible neurophysiological inhibition associated with metal-induced oxidative stress. Conversely, ALP activity correlated positively with Ca (r = 0.6481) and P (r = 0.5927), consistent with its role in phosphorus metabolism, membrane transport, and tissue turnover processes.

Weak or non-significant correlations (|r|≤ 0.50) were observed for most other metal–enzyme pairs, indicating independent biochemical regulation, low-level exposure effects, or complex multivariate interactions. Overall, these patterns suggest that the bioaccumulation of specific elements, notably Fe, Mn, and Zn, may modulate enzymatic responses, although enzyme activities likely reflect the combined influence of metal exposure and intrinsic physiological regulation. Taken together, the correlation coefficients indicate weak to moderate associations between metal concentrations and enzymatic biomarkers (e.g., r = − 0.62 for the Fe–AChE relationship), supporting the interpretation that biochemical responses are driven by multiple concurrent stressors rather than by single-element exposure alone (Figure S1). The integrated chemical and biochemical evidence indicate that fish from the middle Tocantins River are undergoing multi-element exposure capable of eliciting relevant physiological disturbances, with direct implications for ecosystem integrity and human health.

The marked accumulation of As, Pb, and Se, together with alterations in AChE, ALT, AST, and ALP activities, demonstrates that PTEs are not only present but bioavailable and capable of compromising essential metabolic and neurophysiological functions. The two native species evaluated proved to be effective bioindicators due to their ecological relevance, trophic position, abundance, and significant contribution to traditional riverside diets. Their contrasting feeding strategies and habitat use enhance the detection of contaminants originating from both urban effluents and diffuse agricultural sources, allowing a more comprehensive assessment of the basin.

Overall, the integration of chemical and biological evidence indicates that metal contamination in the middle Tocantins River operates across multiple environmental compartments and levels of biological organization. The presence of toxic elements in surface waters and sediments, as documented in previous assessments, is reflected in their accumulation in fish tissues, demonstrating effective transfer from abiotic matrices to aquatic biota. This bioaccumulation is accompanied by consistent sublethal biochemical responses, as evidenced by the modulation of enzymatic biomarkers associated with redox balance, detoxification, and neurochemical regulation. From a One Health perspective, these findings highlight interconnected risks linking environmental degradation, fish health impairment, and human dietary exposure, particularly among vulnerable riverside populations that rely on fish as a primary protein source. Therefore, continuous biomonitoring, improved sanitation infrastructure, and stricter environmental regulation are essential to prevent further ecological degradation and reduce long-term public health risks in the Tocantins River basin.

## Conclusion

This study demonstrates that metal contamination in the middle Tocantins River is not only detectable but biologically active, with implications that extend beyond environmental quality to food safety and public health. The co-occurrence of elevated As and Se concentrations in edible fish tissues with consistent biochemical alterations indicates that these elements are bioavailable and capable of inducing sublethal physiological stress in native species. Importantly, the identification of disproportionate dietary exposure among children under high-consumption scenarios highlights a specific vulnerability that links environmental contamination directly to human health concerns. Rather than representing isolated contamination events, the contrasting patterns observed between urban and rural sites reflect the combined influence of point and diffuse anthropogenic sources within the Cerrado–Amazon ecotone. These results underscore that areas perceived as less impacted may still sustain contaminant levels sufficient to pose chronic risks through food-web transfer. From a management perspective, the findings support the implementation of routine biomonitoring using locally consumed sentinel species, the development of age-specific fish consumption advisories, and the targeted identification and control of As and Se sources within the Tocantins River basin. Framed within a One Health perspective, this work reinforces that the protection of riverine ecosystems is intrinsically linked to food security and community health, providing a scientific basis for informed environmental regulation and public health strategies in one of Brazil’s most socioecologically sensitive regions.

## Supplementary Information

Supplementary Information.

## Data Availability

The data that support the findings of this study are available from the corresponding author upon reasonable request.
