# Supplementary material for: Bioaccumulation of toxic and essential elements and enzymatic responses in native fish from the middle Tocantins River
Source: Sci Rep. 2026 Mar 8;16:12569. doi: 10.1038/s41598-026-39611-3 (PMC13087173; doi:10.1038/s41598-026-39611-3)
Supplement: Supplementary file 1 — Supplementary Information. [file 41598_2026_39611_MOESM1_ESM.docx]

**Figure S1.** Mann–Whitney U test results comparing metal concentrations and enzymatic biomarkers in fish from two sampling sites in the middle Tocantins River, Brazilian Legal Amazon.

CP: Standard length; Weight: Body weight. Al_m: Aluminium (muscle); As_m: Arsenic (muscle); Au_m: Gold (muscle); Ba_m: Barium (muscle); Ca_m: Calcium (muscle); Cr_m: Chromium (muscle); Cu_m: Copper (muscle); Fe_m: Iron (muscle); In_m: Indium (muscle); K_m: Potassium (muscle); Mg_m: Magnesium (muscle); Mn_m: Manganese (muscle); Na_m: Sodium (muscle); Ni_m: Nickel (muscle); P_m: Phosphorus (muscle); S_m: Sulfur (muscle); Sb_m: Antimony (muscle); Se_m: Selenium (muscle); Si_m: Silicon (muscle); Sn_m: Tin (muscle); Zn_m: Zinc (muscle). Al_f: Aluminium (liver); As_f: Arsenic (liver); Au_f: Gold (liver); Ba_f: Barium (liver); Ca_f: Calcium (liver); Co_f: Cobalt (liver); Cu_f: Copper (liver); Fe_f: Iron (liver); K_f: Potassium (liver); Mg_f: Magnesium (liver); Mn_f: Manganese (liver); Ni_f: Nickel (liver); P_f: Phosphorus (liver); Pb_f: Lead (liver); S_f: Sulfur (liver); Sb_f: Antimony (liver); Se_f: Selenium (liver); Si_f: Silicon (liver); Sn_f: Tin (liver); Zn_f: Zinc (liver). AChE_m: Acetylcholinesterase (muscle); ALP_m: Alkaline Phosphatase (muscle); AChE_f: Acetylcholinesterase (liver); AST_f: Aspartate Aminotransferase (liver); ALT_f: Alanine Aminotransferase (liver); ALP_f: Alkaline Phosphatase (liver). Positive values of r (r > 0) indicate direct relationships between variables, whereas negative values (r < 0) indicate inverse relationships. The strength of the associations was interpreted following Akoglu (2018), considering |r| ≤ 0.30 as weak, 0.40–0.60 as moderate, and |r| ≥ 0.70 as strong.
